# The eastern extent of seasonal iron limitation in the high latitude North Atlantic Ocean

**DOI:** 10.1038/s41598-018-37436-3

**Published:** 2019-02-05

**Authors:** A. J. Birchill, N. T. Hartner, K. Kunde, B. Siemering, C. Daniels, D. González-Santana, A. Milne, S. J. Ussher, P. J. Worsfold, K. Leopold, S. C. Painter, M. C. Lohan

**Affiliations:** 1 School of Geography, Earth and Environmental Sciences, Plymouth University, Drake Circus, Plymouth, PL4 8AA United Kingdom; 2 Ocean and Earth Sciences, University of Southampton, Waterfront Campus, National Oceanography Centre, European Way, Southampton, SO14 3ZH United Kingdom; 3 Institute of Analytical and Bioanalytical Chemistry, University of Ulm, Albert-Einstein-Allee 11, 89081 Ulm, Germany; 4 Scottish Association for Marine Science, Scottish Marine Institute, Oban, Argyll PA37 1QA United Kingdom; 5 National Oceanography Centre, European Way, Southampton, SO14 3ZH United Kingdom; 6 Marine Institute, Rinville, Oranmore, Co., Galway, H91 R673, Ireland

## Abstract

The availability of iron (Fe) can seasonally limit phytoplankton growth in the High Latitude North Atlantic (HLNA), greatly reducing the efficiency of the biological carbon pump. However, the spatial extent of seasonal iron limitation is not yet known. We present autumn nutrient and dissolved Fe measurements, combined with microphytoplankton distribution, of waters overlying the Hebridean (Scottish) shelf break. A distinct biogeochemical divide was observed, with Fe deficient surface waters present beyond the shelf break, much further eastwards than previously recognised. Due to along and on-shelf circulation, the Hebridean shelf represents a much-localised source of Fe, which does not fertilise the wider HLNA. Shelf sediments are generally thought to supply large quantities of Fe to overlying waters. However, for this Fe to influence upper-ocean biogeochemical cycling, efficient off-shelf transport mechanisms are required. This work challenges the view that the oceanic surface waters in close proximity to continental margins are iron replete with respect to marine primary production demands.

## Introduction

Marine primary production drives carbon fixation in the ocean and is the base of the marine food web, it is therefore an important component of the Earth system^1^. Iron (Fe) based proteins are required for numerous vital cellular processes (e.g. photosynthesis, respiration, nitrogen fixation), and is therefore an essential nutrient for the growth of marine microbes^2,3^. The solubility of Fe(III), the thermodynamically favored redox species in oxic seawater, is vanishingly low^4^. It is widely accepted that low solubility of Fe, coupled with weak external sources, results in Fe availability regulating phytoplankton primary productivity in the high nutrient low chlorophyll (HNLC) regions of the Southern Ocean, Eastern Equatorial Pacific and Sub-Arctic Pacific^5,6^. The Iceland and Irminger basins of the High Latitude North Atlantic (HLNA) receive comparably low levels of dust input as the sub-Arctic Pacific^7,8^, and exhibit dissolved Fe (dFe) concentrations ranging from 0.02–0.22 nM in surface waters^8–11^. Despite this, it is not considered a classical HNLC region on the basis that there is sufficient Fe available to sustain a productive spring bloom^9,12,13^. However, repeated observations of residual nitrate (NO_3_^−^) in HLNA surface waters after the spring bloom, indicate that there is a definite restriction on the efficiency of the biological carbon pump^10,12,14^.

It is now recognized that seasonal, rather than perennial, Fe limitation following the spring bloom exerts an important control on phytoplankton primary production in the Iceland and Irminger Basins^10,12,15^. Iron is depleted from the surface mixed layer (SML) by uptake and export during the spring bloom^16^, resulting in increased Fe stress as the bloom progresses^12^. Isolation of the SML from deep ocean reservoirs as a result of summer stratification prevents the resupply of Fe from deeper waters^11,17^, causing summer phytoplankton primary production to become Fe limited^10,15^. Much like the Fe limited Southern Ocean^18^, recent Fe budgets for the HLNA show deep winter mixing to be the dominant mechanism of dFe supply to surface waters of the HLNA^11,13^, supplying at least 4–10 times more dFe to surface waters than other Fe sources (atmospheric deposition, vertical diffusive fluxes and horizontal surface fluxes). Therefore, the stoichiometry of this annual winter nutrient flux will largely determine whether complete macronutrient use by phytoplankton can occur during the subsequent spring. Assessments of the nutrient stoichiometry of the winter mixing supply reveal that enough dFe is supplied to surface waters to facilitate drawdown of available silicic acid (Si(OH_4_)), but not NO_3_^−^^11,17^. Consequently, Si(OH)_4_ co-limitation may also exert a control on diatom growth leading to early termination of the spring bloom^19^. In addition to Si(OH)_4_ and Fe limitation, it should be noted that additional factors may contribute to the restriction of NO_3_^−^ uptake in this region, including light limitation of non-siliceous species later in the summer, grazer limitation and phytoplankton species succession leading to dominance of species which preferentially consume recycled nitrogen species (e.g. ammonium)^14^.

**Figure 1:**
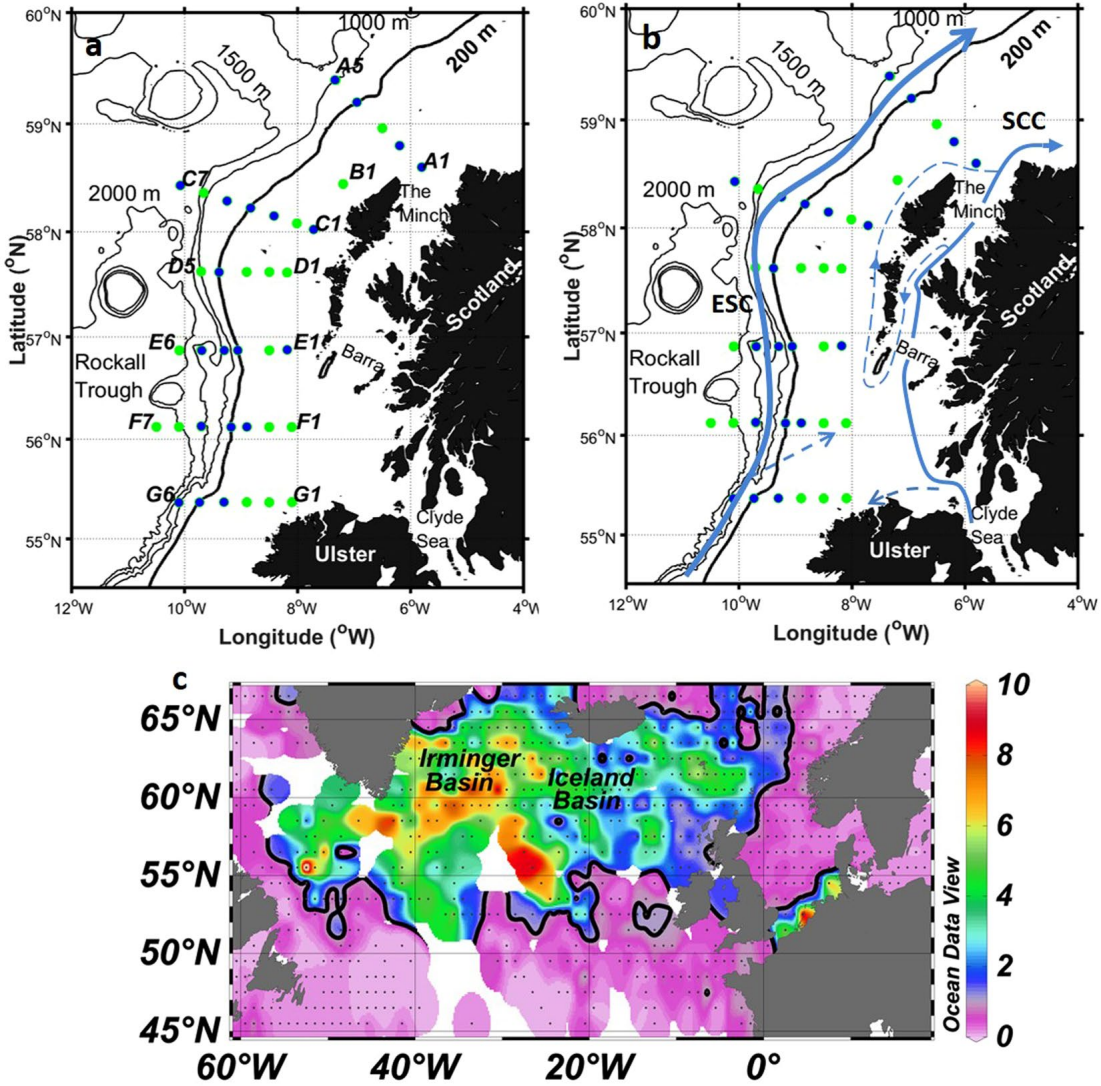
(**A**) Map of survey area denoting CTD sampling stations. Locations which included dissolved iron measurements are indicated by blue fill. (**B**) Map detailing the major currents and their approximate paths (ESC = European Slope Current, SCC = Scottish Coastal Current). (**C**) Climatology of summer surface nitrate concentrations (µM) in the sub-Arctic Atlantic from the World Ocean Atlas^79^. Solid black line indicates the 1 µM contour.

Though process studies have been conducted, and a mechanistic understanding of the processes leading to seasonal Fe limitation in the HLNA developed, the spatial extent of seasonal Fe limitation has not yet been constrained. In this study, the nutrient stoichiometry (dFe: NO_3_^−^: Si(OH_4_): PO_4_^3−^) and microphytoplankton community composition of waters on, and adjacent to, the North West European continental shelf break (hereafter Hebridean Shelf) were determined during Autumn 2014 (Fig. 1a). A detailed description and interpretation of the nutrient and microphytoplankton datasets can be found elsewhere^20,21^. Globally, continental shelf sediments and recycled organic matter are a large source of dFe to the water column^22–26^, with shelf slope (200–2000 m) sediments alone estimated to supply 37 × 10^9^ mol dFe yr^−1^^22^, ~3–4 times larger than the global atmospheric aerosol flux of dissolvable Fe^7,27^. This important source of Fe is known to sustain phytoplankton growth in shelf environments^28–30^ and may also be transported 100–1000’s km into the ocean interior^31–35^. Despite the proximity of the Hebridean Shelf sediments to the HLNA, our results show that seasonal Fe limitation previously observed in the Iceland and Irminger Basins of the HLNA persists up to, and in some instances shoreward of, the Hebridean shelf break. This has therefore led us to identify the Hebridean shelf break as the eastern extent of seasonal Fe limitation in the North Atlantic subpolar gyre. This observation challenges the view that neighboring shelves are prominent sources of Fe to the HLNA.

## Results

### Oceanographic setting and water mass definition

The continental shelf slope separates the Hebridean shelf sea from the Rockall Trough. Steep topography combined with geostrophic forcing, results in a bathymetric steering of flow. This is demonstrated by the Taylor-Proudman theorem, which states that under geostrophic conditions, the steep topography of the shelf slope combined with the effect of the Earth’s rotation, result in a direction of flow over topography that is parallel with (and not across) bathymetric contours. Along the Hebridean shelf slope this is observed as the European Slope Current (ESC; Fig. 1b), a persistent northwards current flowing from the Goban Spur to the Shetland Islands, with seasonal reversals in flow along the Bay of Biscay and Celtic Sea^36–38^. At ~56° N the ESC is predominantly a barotropic flow of ~20 cm s^−1^ parallel to the shelf break, with a characteristic high salinity (35.35–35.40) core at ~200–300 m^38^. The result of near geostrophic conditions is a restriction of cross-shelf exchange. However, frictional forces can violate the assumptions of geostrophy allowing for some cross-shelf exchange of water and material. Over the Hebridean shelf break, this occurs in both surface and bottom waters. At the surface wind driven exchange drives on-shelf flow. In the bottom boundary layer (~100 m thick), frictional forces resulting from the ESC alter the direction of flow so it contains a downslope component, creating a compensating off-shelf downwelling circulation known as the ‘Ekman Drain’. The Ekman Drain transports turbid water at 0.46 m^2^/s, indicating that it is a pathway for off-shelf sediment transport, indeed the Ekman Drain has been shown to rapidly (<10 days) transport diatom chains down the shelf slope to deep waters (>1500 m)^39–42^.

The on-shelf circulation of the Hebridean shelf sea is well studied^43–47^. Near to the coast, the Scottish Coastal Current carries low salinity water originating from the Clyde and Irish Seas around western Scotland and ultimately into the North Sea (Fig. 1b). Flowing northwards, at a mean velocity of ~2–5 cm s^−1^ and volume transport of ~0.1 Sv, the current interacts with the Hebridean islands. The majority (~80%) of flow continues northwards inshore of the Outer Hebridean Islands, the remaining flow returns south through the Minches before flowing northwards along the east coast of the Outer Hebridean Islands^43–46^. Measurements of caesium-137 originating from Sellafield, Cumbria indicate that some outflow from the Irish Sea also occurs westerly along the north coast of Ireland^48^. West of the Scottish Coastal Current, increased salinity (>35) indicates that the shelf water is of Atlantic origin^43,46^. A tongue of high salinity (>35.2) water has been observed penetrating south of the Isle of Barra^46^, though observations at the same location^47^ show that the shoreward extent to which high salinity waters penetrate is interannually variable^43^.

Surface water (upper 700 m) in the Rockall Trough predominantly originates from the Bay of Biscay to the south, but it is also influenced by water entering from the northwest of the basin^49,50^. An important gradient relating to nutrient supply is the shoaling depth of winter convective overturning with decreasing latitude, which reduces the replenishment of surface water nutrients associated with the vertical mixing of intermediate water masses^51^. Consequently, waters originating from south of the Rockall Trough bring less nutrients than waters from the northwest, which are modified by interaction with nutrient rich sub-polar waters in the Iceland Basin^50^.

### Surface iron and macronutrient fields

**Figure 2:**
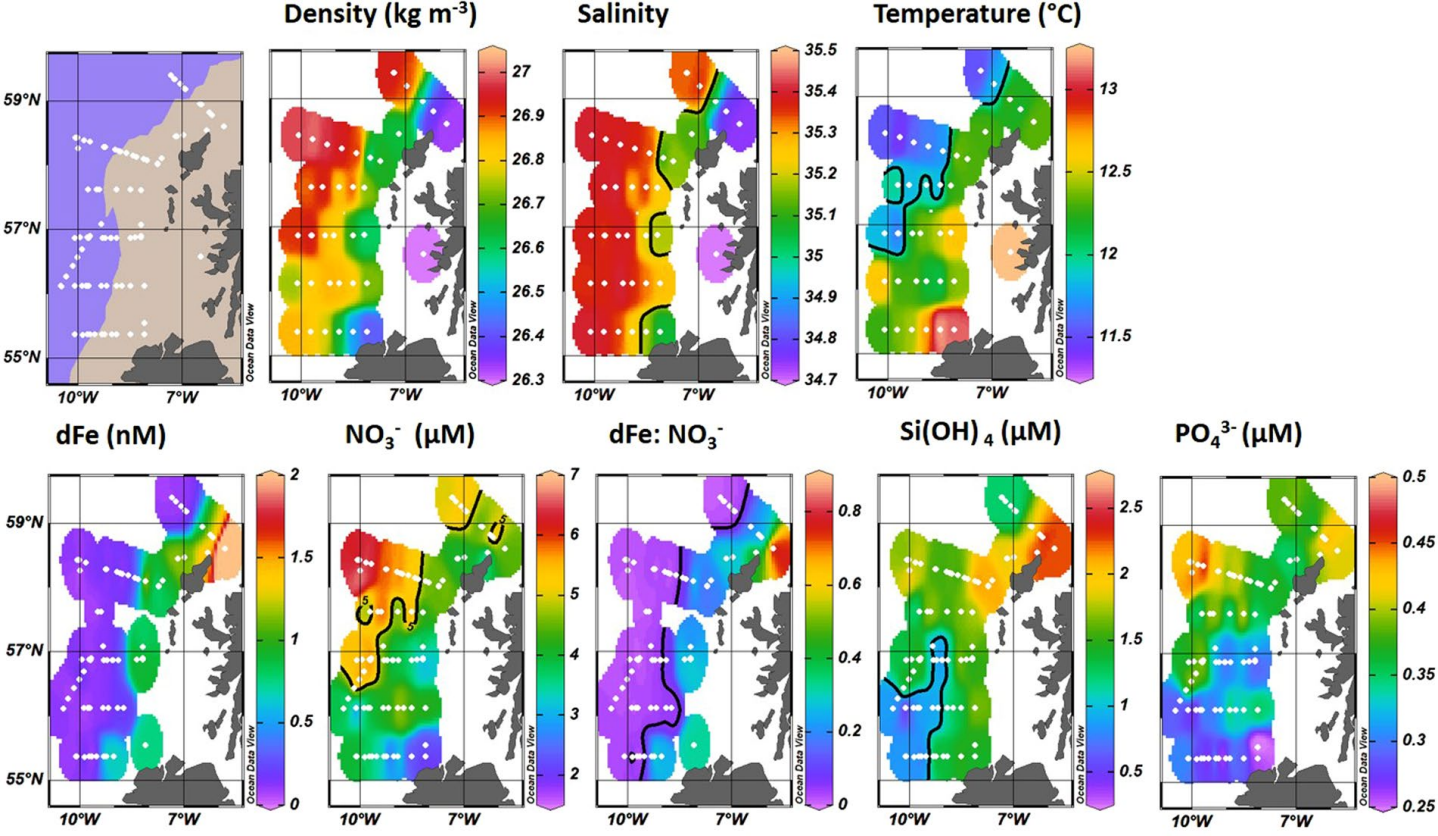
Underway surface maps (as labelled) showing regional gradients in hydrography and nutrient distributions. Brown shaded area on the map indicates bottom depth < 250 m. Solid black lines indicate contours of 35.2 salinity, 11.9 °C, 0.05 dFe:NO_3_^−^, 5 µM NO_3_^−^ and 1 µM Si(OH)_4_. Temperature and salinity data compiled from CTD profiling. Nutrient and dFe data collected from discrete CTD profile samples (~20 m) and underway Tow-fish sampling. (Map details sampling locations of both CTD and Tow-fish data).

The location of dFe depleted HLNA waters were clearly seen in the near-surface water (<20 m) concentrations, where dFe concentrations ranged from 0.03–3.51 nM (Fig. 2). The western (oceanic) extent of the survey area was characterized by elevated salinities (>35.2) and uniformly low dFe concentrations (typically <0.15 nM) (Fig. 2), comparable to concentrations in Fe limited regions^52^. Coincident with the low dFe concentrations were elevated nitrate (NO_3_^−^; 3.22–6.90 µM) and phosphate (PO_4_^3−^; 0.27–0.44 µM) concentrations. The concentrations of NO_3_^−^ and PO_4_^3−^ were higher in the northwest than the southwest of the survey area, with the increase in concentrations associated with cooler surface waters (indicated by the 11.9 °C isotherm; Fig. 2). Silicic acid concentrations were also higher in the northwest than the southwest of the survey area but in both regions remained below 2 µM, which has been suggested as an approximate threshold value for blooms dominated by diatoms^53^. It should be noted however that silicon requirements between species can be highly variable and that diatoms can still grow normally at low Si(OH)_4_ concentrations. For instance, following Fe addition lightly silicified pennate diatoms from the Southern Ocean were able to grow at Si(OH)_4_ concentrations of <1 µM^54^. The north-south gradient in macronutrient distribution over the western (oceanic) extent of the survey area is consistent with surface waters originating from south of the Rockall Trough being relatively nutrient poor in comparison to those entering from the north-west after passage through the Iceland Basin^50^.

Broadly, the salinity of shelf waters decreased and dFe concentration increased towards the coast (Fig. 2), indicating elevated freshwater inputs and isolated water with limited exchange with the open ocean. A number of distinctive mesoscale features were evident in on-shelf surface waters. A salinity minimum (34.7) was observed northeast of the Outer Hebridean Islands, in the path of the Scottish Coastal Current. This was associated with elevated dFe (3.51 nM) and Si(OH)_4_ (2.55 µM) concentrations. A tongue of high salinity (>35.2) Atlantic water penetrated onto the shelf at 56.1° N, consistent with previous observations^46^. This surface water was associated with lower concentrations of dFe (0.21–0.23 nM, *n* = *2*). North of the coast of Ireland, a distinctive tongue of warmer (>13 °C) and fresher (35.1) water was present, consistent with outflow from the Irish Sea^48^. The Irish Sea outflow was characterized by the lowest observed concentrations of NO_3_^−^ (~2 µM) and PO_4_^3−^ (0.25 µM), whilst the concentration of dFe was 0.75 nM.

### Cross-shelf vertical sections of iron and macronutrients

**Figure 3:**
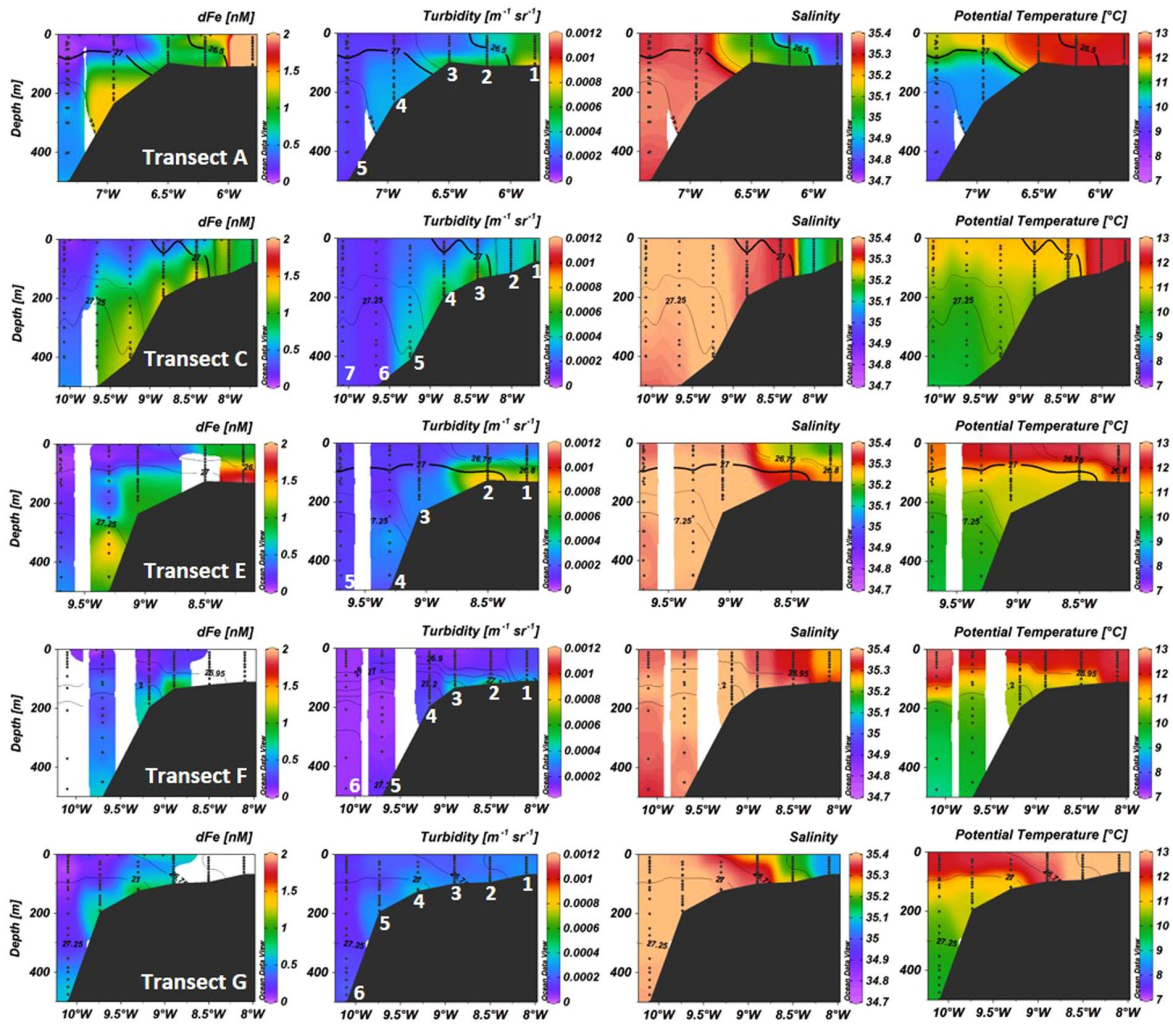
Contoured section plots for the upper 500 m of transects A, C, E, F and G of dFe, turbidity, salinity and potential temperature. Station number identified on the turbidity plot of each transect. Black contour lines represent Sigma-theta (kg m^−3^).

The surface distributions reveal clear cross-shelf and along-shelf gradients in macronutrients and dFe and a very clear divide between shelf and open ocean waters. Due to the non-conservative behavior of dFe in oxic seawater, the concentration of dFe is closely coupled to sources. In this region, the main external sources of dFe are coastal waters and shelf sediments. To illustrate their influence on dFe concentrations, cross-shelf sections of dFe, turbidity, temperature and salinity are plotted in Fig. 3. At the shoreward extent of transect A, the influence of the Scottish Coastal Current is evidenced by low salinity (<34.8) water, with increased particle loading (turbidity) and dFe concentrations >3 nM. However, water with salinities <35.2 was restricted to shallow (<131 m) stations at the eastern (coastal) extent of the cross-shelf sections (Fig. 3). Westward of coastal waters, salinities >35.2 indicated shelf water with an Atlantic source.

Elevated turbidity coincided with increased concentrations of dFe over the shelf sediments (Fig. 3), indicating a sedimentary source of dFe^55^. At stratified on-shelf stations, vertical transport of sedimentary derived dFe to surface waters was restricted by the presence of the seasonal pycnocline (68 ± 24 m, *n* = *12*), consistent with recent work in the nearby Celtic Sea^16^. At the western (oceanic) extent of the survey area, waters with the lowest dFe concentrations (0.03–0.16 nM) were observed above the seasonal pycnocline (79 ± 28 m, *n* = *6*), indicating that lateral transport of dFe from shelf sediments to the surface mixed layer of these stations was limited. This is consistent with the dominant direction of passage for surface waters being either northward in the ESC, or on-shelf as a result of wind driven transport due to prevailing north westerlies^39^. An exception to this was observed at station E4 where an intermediate nepheloid layer was observed at ~300–450 m containing dFe concentrations of 1.17–1.47 nM (Fig. S1). However, this was not observed westward at station E5, indicating that this was a localized feature. Cross-shelf sections of macronutrients are displayed in Fig. S2. At well mixed stations, the trends observed in surface waters were also observed in the vertical distributions. At stratified stations macronutrient concentrations were lower in surface waters and increased at depth (NO_3_^−^ ~12–14 µM, Si(OH)_4_ ~3.5–5.0 µM, PO_4_^3−^ ~0.7–0.9 µM), consistent with uptake in the photic surface waters and release from remineralisation of sinking organic matter^16,56^.

### The European Continental shelf as a boundary between biogeochemical regimes

**Figure 4:**
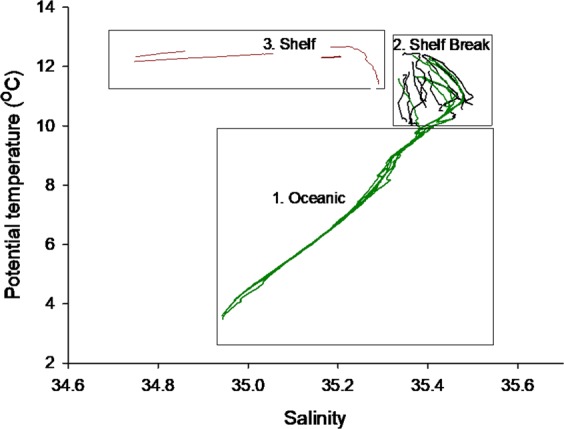
Grouped temperature and salinity plot of stations observed in this study. 1. Shelf stations (red). 2. Surface waters of oceanic stations (green) and stations over the shelf and shelf break (termed shelf influenced) with a corresponding T-S signature (black). 3. Intermediate water masses observed at oceanic stations.

In order to further examine the different oceanographic regimes observed, trace metal sampling stations were classified into three groups based on their temperature-salinity signature (Fig. 4, Table S1). Furthest offshore ‘oceanic’ stations had water column depths of 1013–1865 m, sufficient to observe intermediate water masses (Fig. 4; box 1). At all of these stations salinity increased to >35.40 in the upper 500 m, indicative of the ESC^38^. Stations with a similar T-S signature (T = 10.00–12.40 °C, S = 35.35–35.50; Fig. 4; box 2) were termed ‘shelf break’ and had water column depths of 120–410 m. The remaining stations, with water depths of 76–130 m, were classified as ‘shelf’ stations (Fig. 4; box 3). Shelf stations were fresher than at shelf break stations and oceanic surface waters. However, variations in freshwater input^57^ and the influence of the Scottish Coastal Current resulted in a patchwork of water masses with a wide range of salinities (34.74–35.29). The potential temperature at shelf stations ranged from 11.42–12.67 °C.

The mean concentration of dFe and macronutrients in the SML of each sub-region is displayed in Table 1. The concentrations of dFe and Si(OH)_4_ in the SML of shelf stations were significantly different (p < 0.05) from those observed at shelf break and oceanic stations (Table 1). Therefore, shelf stations were chemically distinct with greater mean dFe (1.62 ± 1.19 nM) and Si(OH)_4_ (4.16 ± 0.88 µM) concentrations.

**Table 1:** Top- The observed mean (±1 standard deviation) nutrient concentrations in the surface mixed layer in October 2014.

	Domain	dFe (nM)	NO_3_^−^ (µM)	Si(OH)_4_ (µM)	PO_4_^3−^ (µM)
Mean surface mixed layer concentrations	Shelf (*n* = 4)	1.62 ± 1.19	4.16 ± 0.88	4.16 ± 0.88	0.37 ± 0.06
	Shelf Break (*n* = 10)	0.18 ± 0.06	5.98 ± 1.13	1.36 ± 0.40	0.36 ± 0.06
	Oceanic (*n* = 5)	0.09 ± 0.01	5.13 ± 0.94	1.28 ± 0.33	0.37 ± 0.06
t-test p value	Shelf vs Oceanic	0.04	**0.95**	0.02	**0.93**
	Shelf vs Shelf Break	0.01	**0.29**	0.02	**0.92**
	Oceanic vs Shelf Break	0.02	**0.95**	**0.35**	**0.83**

Surface mixed layer defined as near surface density plus 0.03 kg m^−3^. Shelf stations A1 and C1 were well mixed. To avoid unequal weighting, the data presented are the mean of individual station means (total individual measurements- shelf stations 19, shelf break 40 and oceanic 19). Bottom- p values from T-tests, p > 0.05 (i.e. not significant) bold. One dFe concentration (0.63 ± 0.01 nM) excluded from the shelf break mean and T-test.

Shelf break and oceanic stations were chemically similar in terms of macronutrient concentrations (Table 1), reflecting the common source of these waters (Fig. 4). In contrast, the concentration of dFe was different (p < 0.05) between oceanic and shelf break stations. Although the dFe concentration was low at shelf break stations (0.18 ± 0.07 nM), it was remarkably low at oceanic stations (0.09 ± 0.01 nM) (Table 1). Additionally, the total dissolvable Fe (TdFe) concentration differed between shelf break (0.62 ± 0.44 nM (*n* = 7)) and oceanic (0.23 ± 0.07 nM (*n* = 7)) stations. These unfiltered samples included both dissolved and particulate Fe.

**Figure 5:**
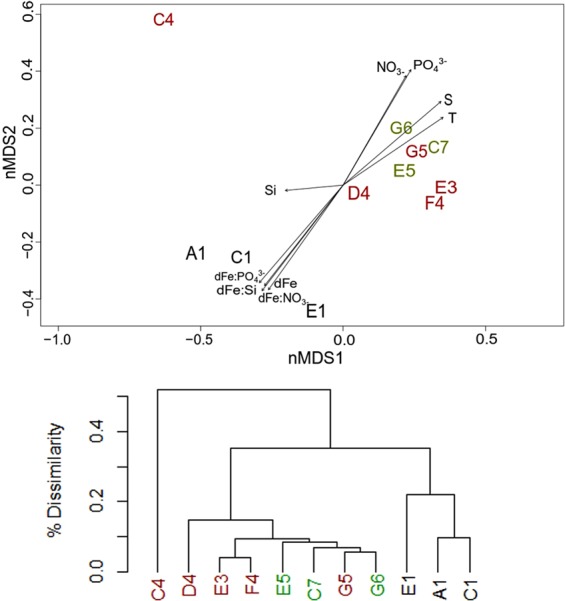
(**A**) Non-metric multidimensional scaling (NMDS) ordination of phytoplankton species at three shelf (black), five shelf break (red) and three oceanic (green) stations. Vectors of environmental factors were plotted on this ordination indicating the relationship between environmental drivers and phytoplankton community composition. The significance of the relationship between an environmental factor and phytoplankton composition is proportional to the length of the vector (T = temperature, S = salinity, NO_3_ ^-^ = nitrate, PO_4_ ^3^^-^= phosphate, Si = silicic acid, Fe = iron, Fe:NO_3_^-^ = iron to nitrate ratio, Fe:PO_4_^3-^ = iron to phosphate ratio, Fe:Si = iron to silicate ratio). (**B**) Dendrogram of the average Bray-Curtis distance between sites and site groupings based on phytoplankton data.

Previous research in this region has demonstrated that contrasting chemical zones between shelf and shelf break/oceanic stations result in distinct microphytoplankton communities^58^. At the time of sampling, the shelf community was dominated by the dinoflagellates *Tripos spp*. or *Prorocentrum spp*. along with the diatom *Pseudo-nitzschia spp*. and the dinoflagellate *Dinophysis spp*. also being present. In contrast, the shelf break/oceanic group was always dominated by only *Tripos spp*, particularly *T*. *fusus*. Species common to shelf stations (*Prorocentrum spp*, *Pseudo-nitzschia spp*. and *Dinophysis spp*) were rare (less than 3% of total count numbers) or absent at shelf break/oceanic stations^20^. To date, the availability of macronutrients and light have been identified as possible environmental drivers of this community divide^20^. To establish the role of environmental drivers in this study, vectors of potential drivers were plotted on top of the ordination plot of species communities at selected stations which had sufficient biological and environmental data available (Fig. 5a). Distance between stations (see Fig. 1 for station names and geographical location) in the ordination plot visualize dissimilarity between biological community at sites, i.e. stations closer together have a higher similarity in terms of phytoplankton assemblage. Percentage dissimilarity was calculated from the Bray-Curtis distance between sites (Fig. 5b). The length of vectors of environmental factors is directly proportional to their significance and the direction of the arrows indicates towards which stations the factor increases. Similar to the findings of Siemering, *et al*.^20^, a study conducted for the same cruise, we found that grouping of microphytoplankton communities (Fig. 5) were significantly related to lower temperatures, salinity, PO_4_^3−^ and NO_3_^−^ concentrations at shelf stations compared to shelf break/oceanic stations (Table 2; p < 0.05). As the availability of Fe can limit phytoplankton growth, we further assessed whether the microphytoplankton community was significantly related to the distribution of dFe concentrations (Fig. 5; Table 2). The vector length and direction in Fig. 5 suggested that higher Fe concentrations and higher Fe: macronutrient ratios at shelf stations relative to shelf break/oceanic stations were significantly related (Table 2; p < 0.05) to the functional grouping of microphytoplankton into shelf and shelf break/oceanic communities, indicating that Fe likely influences the composition of the phytoplankton community.

**Table 2:** Goodness of fit (r^2^) and significance (p-value) of the relationship of environmental drivers to the phytoplankton ordination (Fig. 5) with significance being defined as p ≤ 0.05

Environmental driver	r^2^	P	
PO_4_^3−^	0.8013	0.002	Significant at p < 0.05
Fe: PO_4_^3−^	0.7967	0.006	
NO_3_^−^	0.7046	0.009	
Fe:Si(OH)_4_	0.7372	0.011	
Fe	0.7527	0.017	
Fe:NO_3_^−^	0.7418	0.018	
Salinity	0.7036	0.018	
Temperature	0.5909	0.039	
Si(OH)_4_	0.0926	0.703	Not significant (p > 0.05)

## Discussion

It is clear from these results that the Hebridean Sea represents a transitional zone between oceanic and shelf biogeochemical regimes, with each regime supporting different microphytoplankton communities. The stoichiometry of the autumn nutrient fields observed at oceanic and shelf break stations in this study (Table 1), mirrored that of post spring bloom Fe limited surface waters in the central Iceland basin (dFe < 0.010–0.218 nM, NO_3_^−^ 2–5 µM and Chl-*a* 0.2–0.4 µg L^−1^)^10^. The addition of Fe to bioassays containing Icelandic basin waters resulted in an increase in diatom and coccolithophore abundance and a decrease in NO_3_^−^ concentration^10^. Given the almost identical nutrient concentrations at shelf break/oceanic stations to those observed in the seasonally Fe limited Iceland Basin, we suggest that the biogeochemical transition observed over the Hebridean shelf break is driven, at least in part, by a gradient in Fe stress/limitation. Moreover, it is noteworthy that diatoms were present at all shelf stations (Table S1; 2.5–37.0% of counts) but absent or very rare at all other stations (0.0–0.1% of counts). The restriction of diatom growth is also consistent with Fe limited phytoplankton communities in the HLNA^10,12^.

One possible caveat is that the surface nutrient concentrations observed in October 2014 may not represent the summer minimum due to autumnal mixing and decreasing productivity^20,21^. However, unlike the nearby Celtic Sea^16^, it is known that complete seasonal NO_3_^−^ depletion is not typical, with 0.6 µM of NO_3_^−^ the characteristic summer minimum observed for surface waters during summer (August)^56^. Moreover, even if recent vertical mixing had elevated macronutrients concentrations in surface waters prior to our study, it did not significantly elevate dFe concentrations at shelf break or oceanic stations (Table 1) or result in a change in the microphytoplankton communities.

As sinking organic matter is remineralised it releases macronutrients and dFe back to the water column. This happens at different rates, in the order of P > N > Si^59^, with Fe remineralization thought to be slower than that of P and similar to Si^60^. Additionally, remineralized dFe can be lost to particle formation and scavenging^60^, and in shelf waters NO_3_^−^ is lost via denitrification, eventually becoming dinitrogen^57,59^. These processes shape the vertical profiles of these elements, influencing the stoichiometric ratio at which they are returned to surface waters by vertical mixing and diffusion processes^11,18^.

**Figure 6:**
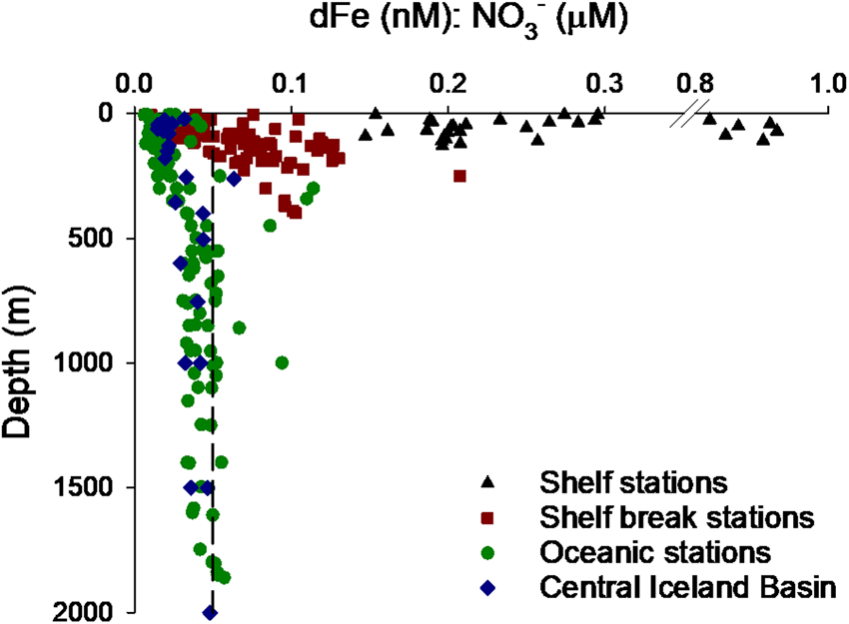
Depth profile of dFe:NO_3_^−^ stoichiometry for each sub-region in this study and for the central Iceland Basin (60.0–60.8 °N, 20.0–21.7 °W)^11^. Dashed line denotes 0.05 dFe:NO_3_^−^ (nM:µM), the lower limit observed in Fe replete cultured phytoplankton^61,62,80^. All samples to right of break in the x axis were from station A1 in the path of the Scottish Coastal Current.

To examine the potential for resupply of nutrients at different rates, depth profiles of dFe:NO_3_^−^ are displayed for shelf, shelf break and oceanic stations and the central Iceland basin in Fig. 6. A cross shelf gradient of dFe:NO_3_^−^ was observed with highest values at shelf stations, a consequence of sediment Fe input and denitrification in shelf sediments^57,59^. Despite very close proximity to the Hebridean shelf slope, and in accordance with profiles from the HLNA^10,11^, the dFe:NO_3_^−^ ratio at oceanic stations was typically less than the cellular Fe content of phytoplankton grown under nutrient replete environments (Fe:N of 0.05–0.9 nM:µM^61,62^) to depths of at least 1800 m (maximum depth of sampling).

**Figure 7:**
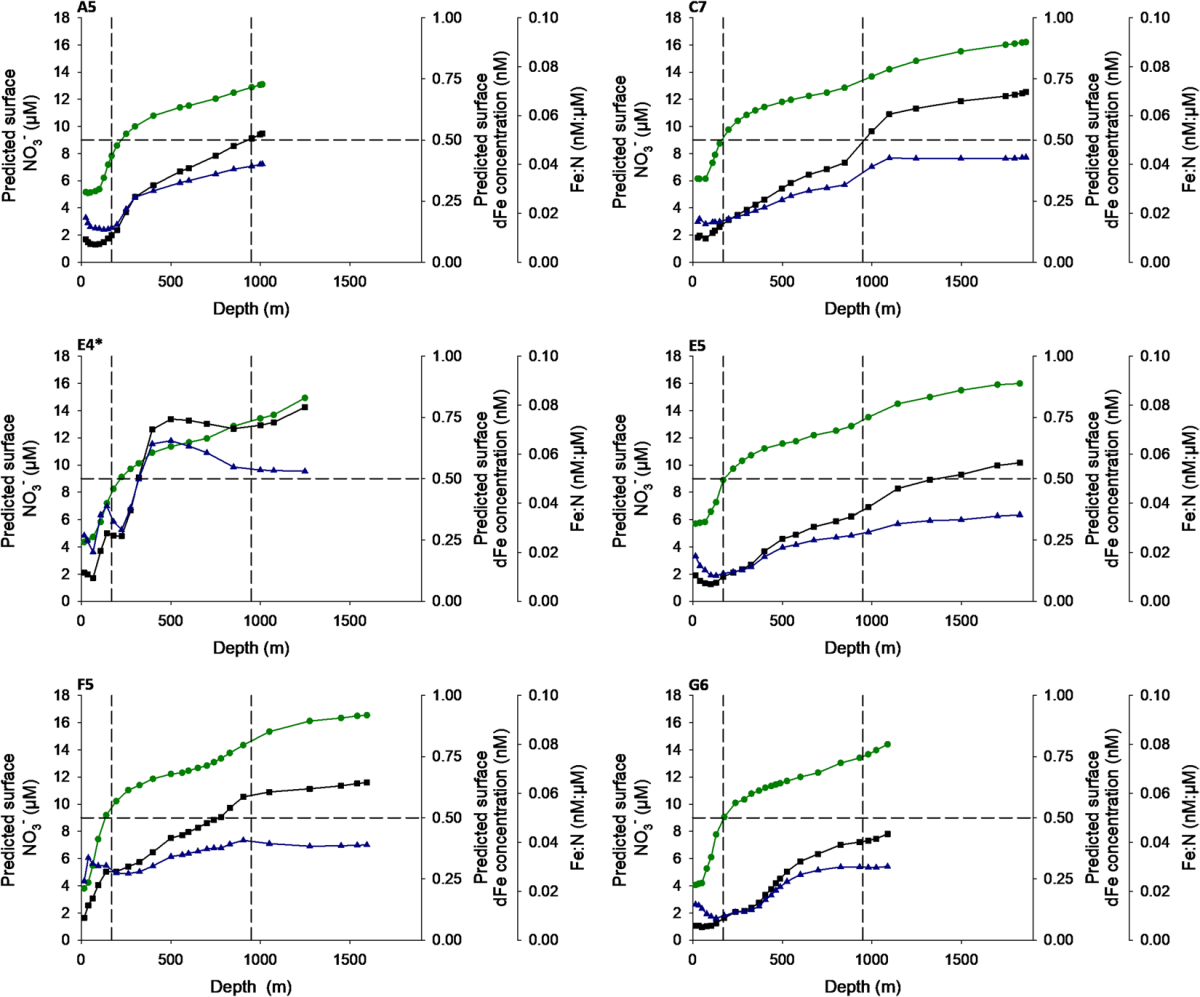
The estimated dFe:NO_3_^−^ surface stoichiometry resulting from winter mixing, calculated by integrating observed autumn concentrations from the surface to each depth sampled. This assumes a closed 1-dimension system. Vertical dashed lines provide an indication of the minimum and maximum winter mixing depths^11,39^. Observed winter (February) NO_3_^−^ concentrations over the Hebridean shelf break are 10–11 µM^57^ and spring (April/May) dFe concentrations are 0.16–0.64 nM^15^. Horizontal dashed line indicates an Fe:N ratio of 0.05 nM:µM, the lowest cellular content of phytoplankton observed during growth nutrient replete cultures^61,62^. ^*^Shallow, iron rich intermediate nepheloid layer observed at station E4 results in an elevated dFe:NO_3_^−^ ratio (see Fig. S1).

Winter mixing homogenises the upper water column, replenishing surface waters with nutrients. This process determines the concentration of nutrients available for the spring bloom and sets a quasi-limit on annual primary production^57^. Based on calculations using our autumn observations and realistic winter mixing depth ranges, it is predicted that winter mixing will consistently result in Fe deficient spring surface waters at oceanic stations (Fig. 7), in the same manner as much of the HLNA^11,17^. This annual process thus preconditions the system towards seasonal Fe limitation, providing an explanation for observations of residual summer nitrate over the Hebridean shelf break. Moreover, much like observations from the Southern Ocean^18^, the sub-surface NO_3_^−^ and dFe pools were decoupled. Both dFe and NO_3_^−^ increased immediately below the seasonal pycnocline, but dFe concentrations increased most significantly at greater depths, presumably due to longer remineralization length scales^60^ and/or mid depth scavenging of dFe^63^. This did not occur as a distinct ferricline but nevertheless drove an increase in the dFe:NO_3_^−^ ratio with depth (Fig. 7). This has two important implications. Firstly, winter mixing depths in the open waters of the HLNA are known to range over several hundred meters, both interannually and spatially^11^. Hence, spring surface waters following a winter of shallower mixing will be more Fe deficient than those following a period of deeper winter mixing. Secondly, nutrients are also supplied to surface waters via a turbulent diffusive flux through the seasonal pycnocline. A comparison of the nutrient stoichiometry immediately above and below the seasonal pycnocline revealed no significant change (p < 0.05) in the dFe:NO_3_^−^ ratio (Fig. S3). Therefore, regardless of the magnitude of this diffusive flux, it will only serve to maintain the observed Fe deficiency in surface waters.

The HLNA is surrounded by land masses and initial investigations into Fe cycling in this region assumed that supply from surrounding land masses represented an important source of Fe^9^. In contrast, our results suggest that the onset of seasonal Fe limitation occurs at the Hebridean shelf break thus excluding this shelf as a source of Fe to the wider HLNA. Recent work has demonstrated that the flux of dissolved inorganic carbon from the Hebridean shelf break occurs by strong downwelling circulation over the shelf break known as the ‘Ekman Drain’^40^. Using water column dFe concentrations combined with Acoustic Doppler Current Profiler measurements, and following the approach of Painter, *et al*.^40^, we estimate a mean shelf break sedimentary Fe flux of 1.45 ± 0.86 µmol dFe m^−2^ d^−1^ (see supporting information). This is similar to, but lower than, late summer diffusive Fe(II) fluxes (3.6–3.9 µmol dFe m^−2^ d^−1^) from Celtic Sea shelf sediments^26^. Dale, *et al*.^22^ recently revised the global dFe flux from shelf sediments by deriving an empirical function relating the benthic organic carbon oxidation rate and bottom water oxygen concentration to sedimentary dFe flux. Combining this function with benthic organic carbon oxidation rates from nearby Goban Spur shelf break (1.5–4.3 mmol C m^−2^ d^−1^)^64^ and bottom water oxygen concentrations from our shelf break profiles (245.8–265.0 µM), results in a calculated flux of 1.0–2.9 µmol dFe m^−2^ d^−1^, comparable to our estimate. Therefore, our results are not in disagreement with shelf slope sediments supplying 37 × 10^9^ mol dFe yr^−1^ globally^22^. However, it is evident from our results that the dFe flux from shelf slope sediments is not necessarily an ‘effective’ flux, in that it does not reach the nearby surface ocean to sustain primary production. Moreover, our Ekman Drain estimate represents the flux of dFe past a point in time and does not account for the particle reactive nature of dFe. In turbid shelf slope waters the rate of loss of dFe to the particulate phase via scavenging is higher than in less turbid open ocean waters^65,66^.

Our observations improve our understanding of Fe cycling in the HLNA. In addition, this work is also a case study demonstrating the ineffectiveness of the shelf derived Fe flux where strong cross-shelf physical transport mechanisms are not present. The circulation over the Hebridean shelf slope presents several obstacles to an effective sedimentary dFe flux to the surface ocean. A restriction is imposed on vertical transport by the presence of the seasonal pycnocline, whilst lateral transport of surface water is predominantly on to or along the shelf with off-shelf advection occurring in bottom waters as the compensating Ekman Drain^38–40,42^. Analogies can be made with other shelf regions. The Greenland shelf is reported to provide the largest sustained input of dFe to the Irminger Basin surface waters (321 nmol m^−2^ d^−1^)^13^. But crucially, the southward flowing East Greenland Current prevents zonal transport beyond the shelf where this dFe could alleviate the observed seasonal Fe limitation^13,67^. Off west Africa, a region where Fe (co)limitation of phytoplankton growth has been observed^68^, convergence of the southward flowing Angola Current with the northwards flowing Benguela Coastal Current at 14.75°S, 12.2°E drives off shelf zonal transport of dFe 100’s km from the West African margin into the South Atlantic^65^. In contrast, at 25°S the northwards flowing Bengula Coastal Current restricts zonal transport of dFe from shelf waters^65^. Finally off New Zealand, the northwards flowing along slope Southland Current has been identified as restricting the lateral transport of shelf derived dFe to the adjacent ocean^69^. Therefore, whilst recent estimates indicate that the global magnitude of shelf sediment dFe flux is far larger than previously considered^22,23,26^, the degree to which this dFe flux impacts upon upper ocean biogeochemical cycling is critically dependent on strong cross-shelf physical transport mechanisms. Where such a mechanism is absent, the relative importance of shelf-derived dFe in sustaining primary production is reduced, allowing other Fe supply processes such as vertical mixing and atmospheric deposition to increase in importance in close proximity to the shelf.

In summary, our work shows how the absence of a direct cross-shelf physical transport mechanism in surface waters over the Hebridean shelf slope means that the conditions for seasonal Fe limitation observed in the HLNA extends ≈ 600 km further eastwards than previously demonstrated^9^. This provides an explanation for the observations of residual summer nitrate over a wide area of the HLNA (Fig. 1c). The mean summer minimum NO_3_^−^ concentration over the Hebridean shelf break is 0.6 ± 0.7 µM^21^. The standard deviation indicates that there exists inter-annual variability in the summer nitrate concentration. This is in contrast to the nearby seasonally stratified regions of the Celtic Sea, where undetectable nitrate concentrations are typical of summer months^70,71^ We cannot identify the driver(s) of this variability, however inter-annual variability of macronutrient concentration of waters in the Rockall Trough has been linked to the retraction of the sub-polar gyre^72^. Therefore, inter-annual variation may be driven in part by changing regional circulation patterns. The hydrography of the north west European shelf is predicted to change over the coming century as a result of climate change, with the strength and length of seasonal stratification expected to increase^73^. This will likely further exacerbate seasonal Fe depletion in surface waters, and thus the severity and spatial extent of seasonal Fe stress may increase.

## Methods

The Hebridean shelf (∼55–60°N, ∼6–10°W) was sampled on-board the *R*.*R*.*S*. *Discovery* in October and November 2014. Six cross shelf transects were conducted from the shelf to the open North East Atlantic Ocean (Fig. 1a). Water samples were collected from 5–24 depths for a range of analyses including for the determination dissolved Fe (dFe), total dissolvable Fe (TdFe), inorganic nutrients, oxygen and microphytoplankton composition.

Salinity, temperature and depth were measured using a CTD system (Seabird 911+), equipped with optical backscatter, dissolved O_2_ and chlorophyll-a sensors which were calibrated daily on-board ship^74^. The determination of macronutrients and oxygen is described in Painter, *et al*.^56^, where these datasets were originally published. Briefly, macronutrient samples were collected in sterile 25 mL plastic vials and analysed immediately using a Skalar San+ segmented autoanalyser, common methodologies (Kirkwood, 1996; Hydes *et al*., 2010) and internationally certified reference materials (http://www.scor-int.org/SCOR_WGs_WG147.htm). Dissolved oxygen concentrations were measured in duplicate with the Winkler whole-bottle titration method^75^. Up to 12 depths were sampled at each station with measurement accuracy estimated to be ± 0.31 μmol L^−1^.

Full details of trace metal sample collection and analysis procedures can be found in Birchill, *et al*.^16^. Briefly, all trace metal samples were collected following GEOTRACES protocols, using a trace metal clean rosette system^76^. Dissolved Fe (0.2 µm filtered) and TdFe (unfiltered) were analysed using flow injection with chemiluminesence detection^77^, after spiking with hydrogen peroxide^78^. The limit of detection (3 x the noise of baseline signal) ranged from 0.02–0.06 nM. The accuracy of the method was evaluated via the analysis of SAFe consensus seawater samples. The concentrations of dFe determined were 0.10 ± 0.03 nM (*n* = *3*) for SAFe S, 0.69 ± 0.05 nM (*n* = *4*) for D1 and 0.95 ± 0.04 nM (*n* = *4*) for SAFe D2. These values are in agreement with published values (S = 0.10 ± 0.008 nM, D1 = 0.67 ± 0.04 nM, D2 = 0.96 ± 0.024 nM). The combined expanded uncertainty was estimated from the daily analysis of in-house standards and periodic analysis of consensus materials using the Nordtest approach. An expanded combined analytical uncertainty of 15.7% (k = 2, approximates to 2 SD) was estimated for dFe concentrations ranging from 0.69–1.59 nM (see supporting information for details).

Additionally, surface seawater was pumped into the trace metal clean laboratory using a Teflon diaphragm pump (Almatec A-15, Germany) connected by acid-washed braided PVC tubing to a towed “fish” positioned at approximately 2–3 m depth using a davit on the port side of the ship. Underway samples for the determination of dFe were filtered in-line through 0.2 µm pore size acetate membrane filter capsules (Sartobran-P size 7, Sartorius).

A total of 11 stations were sampled for both dissolved Fe and microphytoplankton (A1, C1, C4, D4, E1, E3, E5, F4, G5, G6). A detailed description of the sampling and analysis procedures of microphytoplankton can be found in Siemering, *et al*.^20^, where this dataset was originally published. In short, phytoplankton cells were collected via a 20 µm phytoplankton net haul and immediately fixed with acidic Lugol’s Iodine solution. Cells were allowed to settle for a minimum of 22 h in 10 mL or 25 mL settling chambers, before being identified under 200x magnification using a Zeiss Axiovert 100 inverted light microscope. Cell counts were calculated as cells L^−1^ to account for differences in total sampled and settled volumes and fourth root transformed to adjust for species with high abundance. The Bray-Curtis distance between phytoplankton communities was used to calculate differences between sites. R vegan functions were used to create the non-metric multidimensional scaling ordination for phytoplankton data to visualize similarities between sites with more similar sites being closer together on the ordination. Environmental data was also fitted onto the ordination to determine the significance of environmental drivers on phytoplankton community similarity, calculated as r^2^ as a measure of goodness of fit for a linear model (see Siemering, *et al*.^20^ for details).

## Supplementary information

Supporting Information
