# Supplementary material for: The eastern extent of seasonal iron limitation in the high latitude North Atlantic Ocean
Source: Sci Rep. 2019 Feb 5;9:1435. doi: 10.1038/s41598-018-37436-3 (PMC6363741; doi:10.1038/s41598-018-37436-3)
Supplement: Supplementary file 1 — Supporting Information [file 41598_2018_37436_MOESM1_ESM.pdf]

# The eastern extent of seasonal iron limitation in the high latitude North Atlantic Ocean

**A. J. Birchill[1,5*], N. T. Hartner[1,3], K. Kunde[2], B. Siemering[4,6], C. Daniels[5], D. González-Santana[2], A. Milne[1], S. J. Ussher[1], P.J. Worsfold[1], K. Leopold[3], S. C. Painter[5], M. C. Lohan[1,2]**

[1] School of Geography, Earth and Environmental Sciences, Plymouth University, Drake Circus, Plymouth, PL4 8AA, United Kingdom.

[2] Ocean and Earth Sciences, University of Southampton, Waterfront Campus, National Oceanography Centre, European Way, Southampton, SO14 3ZH.

[3] Institute of Analytical and Bioanalytical Chemistry, University of Ulm, Albert-Einstein-Allee 11, 89081 Ulm.

[4] Scottish Association for Marine Science, Scottish Marine Institute, Oban, Argyll, PA37 1QA.

[5] National Oceanography Centre, European Way, Southampton, SO14 3ZH.

[6] Marine Institute, Rinville, Oranmore, Co. Galway, Ireland.

Corresponding author: Antony Birchill (anchill@noc.ac.uk)

**Station Classification**

**Table S1-** Trace metal sampling stations classified into 3 different sub regimes based on their temperature and salinity signature; Shelf, Shelf Break and Oceanic.

| Station | Longitude (°W) | Latitude (°N) | Bottom depth (m) | Domain |
|---|---|---|---|---|
| A1 | -5.803 | 58.602 | 110 | Shelf |
| A2 | -6.195 | 58.801 | 112 | Shelf |
| C1 | -7.716 | 58.023 | 76 | Shelf |
| E1 | -8.183 | 56.875 | 130 | Shelf |
| A4 | -6.949 | 59.196 | 225 | Shelf break |
| C3 | -6.418 | 58.149 | 136 | Shelf break |
| C4 | -8.832 | 58.223 | 193 | Shelf break |
| C5 | -9.248 | 58.289 | 401 | Shelf break |
| D4 | -9.391 | 57.62 | 191 | Shelf break |
| E3 | -9.059 | 56.88 | 191 | Shelf break |
| F3 | -8.898 | 56.122 | 128 | Shelf break |
| F4 | -9.177 | 56.119 | 187 | Shelf break |
| G4 | -9.302 | 55.371 | 120 | Shelf break |
| G5 | -9.736 | 55.369 | 191 | Shelf break |
| A5 | -7.339 | 59.398 | 1013 | Oceanic |
| C7 | -10.077 | 58.433 | 1865 | Oceanic |
| E4 | -9.299 | 56.87 | 1399 | Oceanic |
| E5 | -9.696 | 56.869 | 1851 | Oceanic |
| F5 | -9.704 | 56.128 | 1612 | Oceanic |
| G6 | -10.101 | 55.367 | 1130 | Oceanic |

## Intermediate nepheloid layer at station E4

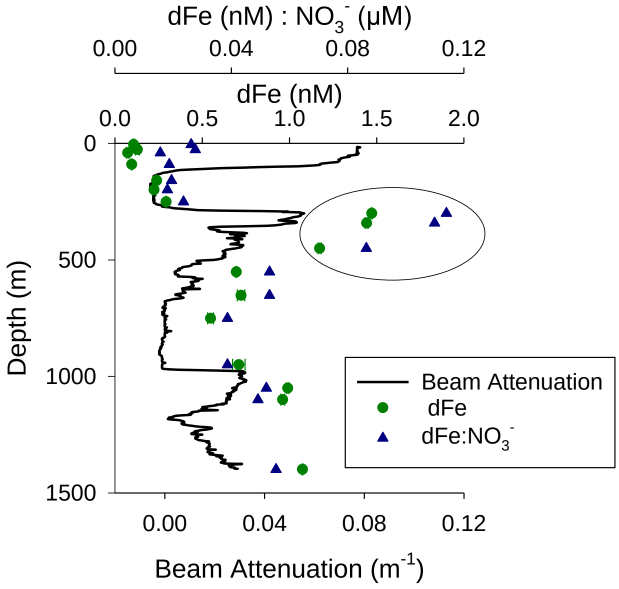


**Figure S1-** The impact of nepheloid layers on the depth profile of dFe, dFe: $NO_3^-$ and beam attenuation at station E4. Oval encompasses samples collected from shallow nepheloid layer(s).

## Cross shelf sections of macronutrients

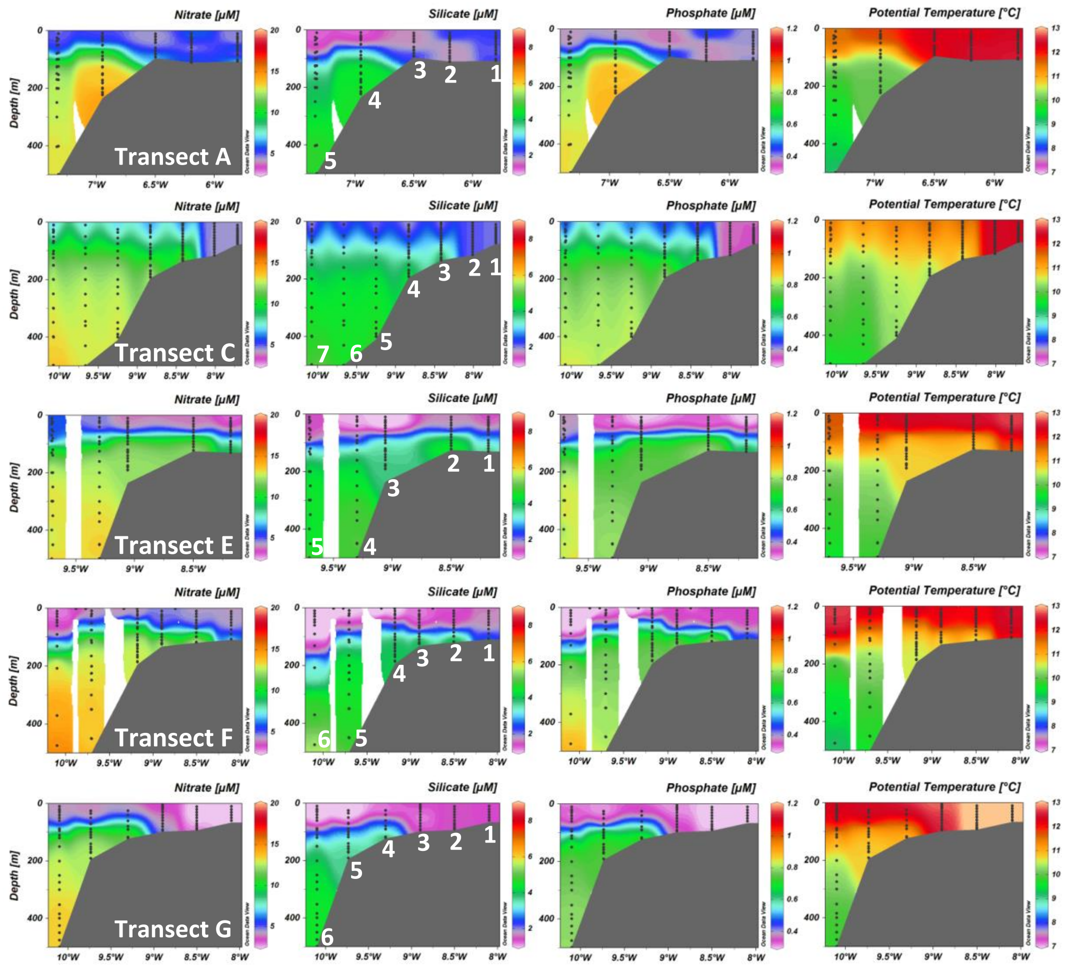


**Figure S2**- Contoured section plots for the upper 500m of transects A, C, E, F and G of, Nitrate ($NO_3^-$), Silicate ($Si(OH)_4$), Phosphate ($PO_4^{3-}$) and potential temperature. Within each transect station number identified on silicate plot.

**Stoichiometry of the diffusive flux through the pycnocline**

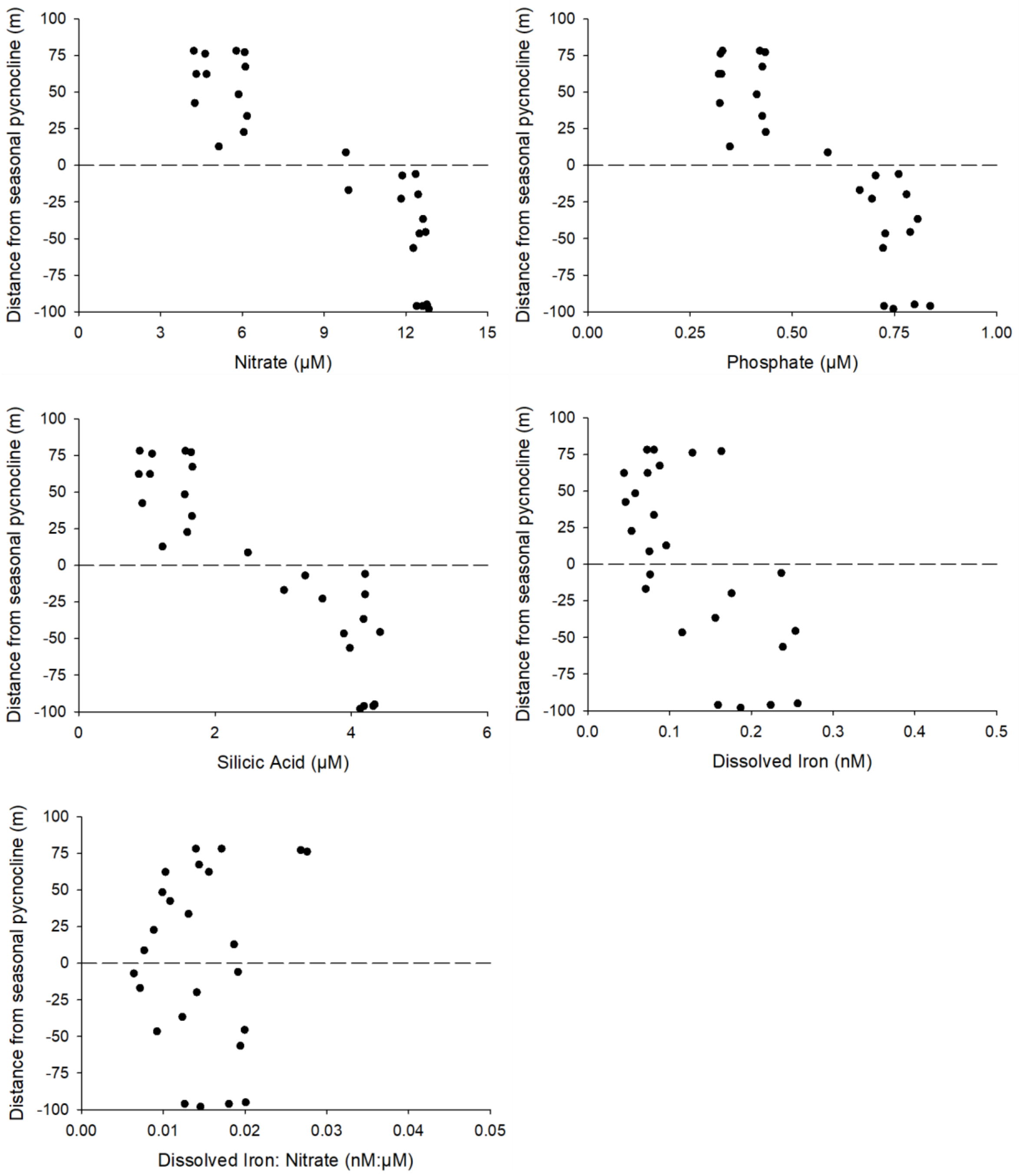


**Figure S3**- The observed nutrient stoichiometry above and below the seasonal pycnocline at oceanic stations. Dashed line indicates location of the seasonal pycnocline. The concentration of all nutrients (including dFe) increased below the pycnocline (Mann-Whitney Rank sum test for non-normally distributed nitrate ($p < 0.001$), phosphate ($p < 0.001$) and dissolved iron ($p = 0.001$), t-test for normally distributed silicic acid ($p < 0.001$). There was no statistical difference in the dissolved iron to nitrate ratio above or below the pycnocline (t-test $p = 0.8$).

**Sediment flux estimations**

The regional dFe flux was estimated following Painter, et al. [1]. Briefly, fluxes were estimated for a region covering 55.3–59.2°N, and from the 1000 m contour as the western boundary to a nominal inshore eastern boundary at 7.8°W (Figure 1). This region covers ~54,747 $km^2$, and represents approximately 5% of the total area of the NW European Shelf (1112 × 103 $km^2$). The length of the shelf edge in the study area (distance along the 200 m contour) is 516 km.

Transport was estimated using 75 and 150 kHz RDI "Ocean Surveyor" Acoustic Doppler Current profilers (ADCP). We preferentially used the 75 kHz instrument due to its deeper acoustic penetration and during the cruise, this was set to operate in "narrowband" mode and to average over 120 s intervals with 60 depth bins of 16 m thickness with data acquisition via VM-DAS. Preliminary data processing at sea corrected for instrument mis-alignment angle and vessel motion as described in best-practice guidelines.[2]

The values used to derive regional shelf edge dFe estimates are displayed in Table S2. The dFe flux (mol $m^{-2}$ $d^{-1}$) was calculated by multiplying water transport by the concentration of dFe in bottom waters over the shelf break (station nearest the 200 m contour) for each transect (Equ.1). The total shelf edge flux was estimated by multiplying this by the length of the shelf edge covered in the study (Equ.2). This was converted to a flux per unit area by dividing the shelf edge flux by the area of the Hebridean shelf (Equ.3).

Equ.1 dFe flux (mol $m^{-2}$ $d^{-1}$) = (transport ($m^2$ $s^{-1}$) x ([dFe] (µmol $m^{-3}$) / 1000000)) x 86400 s

Equ.2 Shelf edge dFe flux (mol dFe $d^{-1}$) = dFe flux (mol $m^{-2}$ $d^{-1}$) x 516000 m

Equ.3 dFe flux per unit area (µmol dFe $m^{-2}$ $d^{-1}$) = (shelf edge flux (mol dFe $d^{-1}$) x 1000000) / (54747 $km^{-2}$ x 1000000)

**Table S2-** The observed and calculated values used to derive regional shelf dFe flux estimates.

| **Transect** | **Transport ($m^2 s^{-1}$)** | **dFe concentration (μmol $m^{-3}$)** | **dFe flux (mol $m^{-2} d^{-1}$)** | **Shelf edge flux (mol dFe $d^{-1}$)** | **dFe flux per unit area (μmol dFe $m^{-2} d^{-1}$)** |
|---|---|---|---|---|---|
| A | 1.56 | 1.49 | 0.201 | 103627 | 1.89 |
| C | 0.46 | 1.79 | 0.071 | 36709 | 0.67 |
| D | 2.68 | 1.16 | 0.269 | 138598 | 2.53 |
| E | 2.5 | 1.07 | 0.231 | 119258 | 2.18 |
| F | 1.57 | 0.66 | 0.090 | 46196 | 0.84 |
| G | 0.74 | 1.01 | 0.065 | 33321 | 0.61 |
| Mean | 1.81 | 1.2 | 0.188 | 96832 | 1.45 |
| SD | 0.90 | 0.39 | 0.090 | 46320 | 0.85 |

## Nordtest estimation for analytical uncertainty

The Nordest[3] approach incorporates within laboratory reproducibility over a period several months of analyses and also accounts for systematic bias. It is therefore a more realistic estimate than the short-term reproducibility of replicate analyses of a single sample. In this case, the analysis of two in-house quality control standards and SAFe D1 and D2 consensus materials were used to estimate combined uncertainty for analyses conducted over 5 months (Table S3). The combined expanded uncertainty (approximates to 2 SD) was estimated as 15.7% for dFe concentrations ranging 0.69-1.59 nM. This is larger than analytical uncertainties typically reported in oceanic trace metal measurements where the short-term analytical reproducibility is used. It is however comparable to a top down combined uncertainty estimate (10-15%) for the same analytical approach for dFe concentrations ranging 0.5-1.0 nM.[4] It is also comparable to combined expanded uncertainties of 13-25%, also calculated using the Nordtest approach, reported for automated preconcentration of Fe, Zn, Cu, Ni, Cd, Pb, Co and Mn in seawater followed by analysis with high-resolution sector field inductively-coupled plasma mass spectrometry.[5] We advocate that these higher analytical uncertainties represent a more realistic estimate, and therefore future oceanic trace metal studies should consider this approach. In our study, dFe concentrations ranged from $< 0.1$ nM to $> 3.0$ nM, which is much larger than the estimated combined expanded uncertainty.

**Table S3**- Materials used in the combined expanded uncertainty estimate. Materials CS #1 and CS #2 are in house quality control materials, which were filtered (0.2 µm) samples collected from the Celtic Sea.

| | CS #1 | CS #2 | SAFe D1 | SAFe D2 |
|---|---|---|---|---|
| Mean Measured Concentration (nM) (± SD) | 1.26 (0.09) | 1.59 (0.08) | 0.69 (0.05) | 0.95 (0.05) |
| n | 26 | 17 | 4 | 4 |
| Consensus value (nM) (± SD) | n.a | n.a | 0.69 (0.04) | 0.956 (0.024) |

## References


1 Painter, S. C. *et al.* Carbon exchange between a shelf sea and the ocean: The Hebrides Shelf, west of Scotland. *Journal of Geophysical Research: Oceans* **121**, 4522-4544 (2016).

2 Firing, E. & Hummon , J., M. Shipboard ADCP measurements. The GO-Ship Repeat Hydrography Manual: A collection of expert reports and guidelines. *IOCCP Rep. 14* **1**, 1-11, doi: http://www.go-ship.org/HydroMan.html (2010).

3 Magnusson, B., Näykki, T., Hovind, H., Krysell, M. & Sahlin, E. *Handbook for Calculation of Measurement Uncertainty in Environmental Laboratories*. 4 edn, (Nordtest Report TR 537, 2017).

4 Floor, G. H. *et al.* Combined uncertainty estimation for the determination of the dissolved iron amount content in seawater using flow injection with chemiluminescence detection. *Limnology and Oceanography: Methods* **13**, 673-686 (2015).

5 Rapp, I., Schlosser, C., Rusiecka, D., Gledhill, M. & Achterberg, E. P. Automated preconcentration of Fe, Zn, Cu, Ni, Cd, Pb, Co, and Mn in seawater with analysis using high-resolution sector field inductively-coupled plasma mass spectrometry. *Analytica chimica acta* **976**, 1-13 (2017).
